# An IFNγ/CXCL2 regulatory pathway determines lesion localization during EAE

**DOI:** 10.1186/s12974-018-1237-y

**Published:** 2018-07-16

**Authors:** Joshua S. Stoolman, Patrick C. Duncker, Amanda K. Huber, David A. Giles, Jesse M. Washnock-Schmid, Athena M. Soulika, Benjamin M. Segal

**Affiliations:** 1 Holtom-Garrett Program in Neuroimmunology and Multiple Sclerosis Center, Department of Neurology, University of Michigan School of Medicine, Ann Arbor, MI 48109 USA; 2 Graduate Program in Immunology, University of Michigan School of Medicine, Ann Arbor, MI 48109 USA; 3 Graduate Program in Neuroscience, University of Michigan School of Medicine, Ann Arbor, MI 48109 USA; 4 Institute for Pediatric Regenerative Medicine, UC Davis School of Medicine and Shriners Hospital, 2425 Stockton Blvd, Sacramento, CA 95817 USA; 5 Neurology Service, VA Ann Arbor Health Care System, Ann Arbor, MI USA; 6 Division of Allergy-Immunology, Division of Pulmonary and Critical Care, Northwestern University, Feinberg School of Medicine, 240 E. Huron Street, McGaw M410, Chicago, IL 60611 USA

**Keywords:** Experimental autoimmune encephalomyelitis, Multiple sclerosis, Chemokines, Cytokines, Neutrophils, Monocytes, Interferon-gamma

## Abstract

**Background:**

Myelin oligodendrocyte glycoprotein (MOG)-reactive T-helper (Th)1 cells induce conventional experimental autoimmune encephalomyelitis (cEAE), characterized by ascending paralysis and monocyte-predominant spinal cord infiltrates, in C57BL/6 wildtype (WT) hosts. The same T cells induce an atypical form of EAE (aEAE), characterized by ataxia and neutrophil-predominant brainstem infiltrates, in syngeneic IFNγ receptor (IFNγR)-deficient hosts. Production of ELR+ CXC chemokines within the CNS is required for the development of aEAE, but not cEAE. The cellular source(s) and localization of ELR+ CXC chemokines in the CNS and the IFNγ-dependent pathways that regulate their production remain to be elucidated.

**Methods:**

The spatial distribution of inflammatory lesions and CNS expression of the ELR+ CXC chemokines, CXCL1 and CXCL2, were determined via immunohistochemistry and/or in situ hybridization. Levels of CXCL1 and CXCL2, and their cognate receptor CXCR2, were measured in/on leukocyte subsets by flow cytometric and quantitative PCR (qPCR) analysis. Bone marrow neutrophils and macrophages were cultured with inflammatory stimuli in vitro prior to measurement of CXCL2 and CXCR2 by qPCR or flow cytometry.

**Results:**

CNS-infiltrating neutrophils and monocytes, and resident microglia, are a prominent source of CXCL2 in the brainstem of IFNγRKO adoptive transfer recipients during aEAE. In WT transfer recipients, IFNγ directly suppresses CXCL2 transcription in microglia and myeloid cells, and CXCR2 transcription in CNS-infiltrating neutrophils. Consequently, infiltration of the brainstem parenchyma from the adjacent meninges is blocked during cEAE. CXCL2 directly stimulates its own expression in cultured neutrophils, which is enhanced by IL-1 and suppressed by IFNγ.

**Conclusions:**

We provide evidence for an IFNγ-regulated CXCR2/CXCL2 autocrine/paracrine feedback loop in innate immune cells that determines the location of CNS infiltrates during Th1-mediated EAE. When IFNγ signaling is impaired, myeloid cell production of CXCL2 increases, which promotes brainstem inflammation and results in clinical ataxia. IFNγ, produced within the CNS of WT recipients, suppresses myeloid cell CXCR2 and CXCL2 production, thereby skewing the location of neuroinflammatory infiltrates to the spinal cord and the clinical phenotype to an ascending paralysis. These data reveal a novel mechanism by which IFNγ and CXCL2 interact to direct regional recruitment of leukocytes in the CNS, resulting in distinct clinical presentations.

**Electronic supplementary material:**

The online version of this article (10.1186/s12974-018-1237-y) contains supplementary material, which is available to authorized users.

## Background

In multiple sclerosis (MS), a multifocal inflammatory demyelinating disease of the central nervous system (CNS), the distribution of lesions can vary widely between patients, resulting in distinct clinical phenotypes. In some patients, lesion burden is dispersed fairly evenly across CNS compartments, while in others it is skewed either towards the spinal cord or supratentorial white matter [1, 2]. The histopathology of MS lesions, including the presence of specific immune subsets and factors, is also diverse. Similarly, lesion distribution and composition is heterogeneous in experimental autoimmune encephalomyelitis (EAE), which is widely used as an animal model of MS.

Although little is known about the factors that determine the composition or location of lesions in MS, several studies have demonstrated a pivotal role of IFNγ in EAE. Adoptive transfer of Th1-polarized, myelin oligodendrocyte glycoprotein (MOG)-reactive T cells derived from C57BL/6 wildtype (WT) mice into syngeneic wildtype (WT) hosts results in a high incidence of “conventional” EAE (cEAE), which manifests as an ascending paralysis secondary to monocyte-rich inflammatory infiltration of the thoracolumbar spinal cord. Conversely, transfer of the same population of Th1 cells into IFNγ receptor knockout (IFNγRKO) hosts, or transfer of IFNγKO CD4^+^ T cells into WT hosts, results in a high incidence of atypical EAE (aEAE), characterized by gait imbalance and brainstem or cerebellar inflammation [3–5]. IFNγ induces expression of chemokines, such as CCL2 and CXCL10 [6], and endothelial adhesion molecules, such as VCAM-1 and P-selectin [7, 8], in the CNS. Collective induction of these molecules promotes neuroinflammation by facilitating the passage of T cells and monocytes into the CNS parenchymal white matter. In fact, cEAE is dependent on spinal cord expression of CCR2 and α4β1 integrin, the receptors for CCL2 and VCAM-1, respectively [5, 9, 10]. In contrast, aEAE lesion development correlates with production of ELR^+^ CXC chemokines in the brainstem and is neutrophil dependent [5]. The destructive effects of neutrophil infiltration in CNS white matter have been demonstrated in several different animal models [11, 12]. Hence, the relative importance of particular cytokine and chemokine pathways varies across different models of EAE, which can translate into differential responsiveness to individual disease-modifying therapies [3–5, 13, 14].

For the future development of personalized approaches to MS therapy, it will be important to acquire a detailed understanding of the spectrum of molecular and cellular pathways that underlie similar, as well as divergent, clinical courses of autoimmune demyelinating disorders. In the current study, we investigate the cellular source and spatial localization of the ELR^+^ CXC chemokines, CXCL1 and CXCL2, in C57BL/6 IFNγRKO mice with aEAE, the factors that trigger CXCL2 production, and the mechanism by which IFNγ regulates neutrophil infiltration of the brainstem.

## Methods

### Mice

Eight- to 14-week-old CD45.1 congenic and WT C57BL/6 mice were obtained from Charles River/NCI Fredrick. IFNγRKO and Rosa^mT/mG^ mice were originally obtained from Jackson Laboratory and bred in the University of Michigan vivarium. LysM-Cre:IFNγRflox/flox mice were obtained from Athena Soulika, University of California-Davis [15]. All mice were housed in microisolator cages under specific pathogen-free conditions. All animal protocols were approved by the University of Michigan Committee on Use and Care of Animals.

### Antibodies and reagents

For flow cytometry, the following antibodies were obtained from eBioscience: PECy7-α-CD11b (M1/70), eFluor450-α-CD45 (30-F11), and PerCpCy5.5-α-Ly6C (HK1.4). Allophycocyanin cy7-α-Ly6G (IA8) was from BD Biosciences. For immunofluorescent histology, primary antibodies included rabbit α-glial fibrillary acidic protein (GFAP) (Gibco), rat α-mouse CD45 (IBL-5/15, Millipore), goat α-mouse CXCL2 (R&D Systems), goat α-mouse CXCL1 (R&D Systems), rat α-mouse Ly6G (IA8, BD Biosciences), and hamster α-mouse CD3ε (BD Biosciences). All secondary antibodies were from Life Technologies, including AlexaFluor594 donkey-α-goat IgG, AlexaFluor488 goat-α-rat IgG, goat α-hamster, and AlexaFluor647 goat-α-rabbit IgG. For in vitro cultures, G-CSF was obtained from Amgen; recombinant mouse (rm) IFNγ, rmIL-12, rmCXCL2, rmIL-1β, and rmCXCL1 were from R&D Systems.

### Induction and scoring of EAE

Donor mice were immunized subcutaneously with 100 μg MOG_35–55_ (MEVGWYRSP-FSRVVHLYRNGK, Biosynthesis) in CFA (Difco) across four sites over the flanks. Inguinal, axial, and brachial lymph nodes were harvested 14 days post-immunization, pooled, homogenized, and passed through a 70-μm strainer (BD Falcon). Cells were cultured with MOG_35–55_ (50 μg/mL) in the presence of rmIL-12 (6 ng/mL) and rmIFNγ (2 ng/mL). At 96 h, CD4 T cells were isolated by column separation with CD4 (L3T4) magnetic microbeads, according to the manufacturer’s instructions (Miltenyi). CD4 T cells (> 80% pure) were transferred i.p. into naïve hosts (5 × 10^6^ cells/ mouse). Adoptive transfer recipients were monitored on a daily basis by an examiner who was blinded to the experimental groups. Mice were scored for severity of conventional and atypical signs of EAE using established scales [4, 5].

### Construction of bone marrow chimeric mice

Femurs and tibiae of IFNγRKO or ROSA^mT/mG^ mice were flushed with PBS using a 26-gauge needle to obtain donor bone marrow cells. Cells were ACK lysed and suspended in cold PBS for intravenous injection into CD45.1 congenic hosts, which had been subjected to 13 Gy of irradiation (orthovoltage X-ray source) split into two doses, 3 h apart. Hosts were given 2.5 × 10^6^ bone marrow cells from each donor source (1:1 ratio) and allowed to reconstitute for at least 6 weeks before further use. Chimerism was verified by flow cytometric analysis of peripheral blood mononuclear cells.

### Histochemical procedures

Mice were perfused with 1× PBS and 4% paraformaldehyde (PFA). CNS tissues were harvested, post-fixed in 4% PFA for 96 h, decalcified in 0.5 M EDTA for 96 h, and transferred into 30% sucrose for at least 48 h prior to embedding in OCT and storage at − 80 °C. Spinal cord and brain tissues were cryosectioned at 10 μm. Sections were incubated in 1× PBS in a humidified chamber, followed by blocking solution (1× PBS 7.4 pH, 10% Normal Donkey Serum, 0.5% Triton-X100) for 1 h. Primary antibodies were applied overnight at 4 °C. For secondary antibody staining, sections were incubated with AlexaFluor594 donkey-α-Goat IgG, rinsed in PBS, and then incubated with AlexaFluor488 goat-α-rat IgG and AlexaFluor647 goat-α-rabbit IgG followed by DAPI (100 ng/mL). Sections were washed and mounted on slides (Antifade Reagent, Southern Biotech). Confocal images were acquired using a Nikon A-1 confocal microscope (Nikon PlanApoVC × 20, × 40, or × 60/1.40 oil) with diode-based laser system and NIS Elements software.

For in situ hybridization, cryosections of CNS tissue were incubated with Proteinase K (10 μg/mL, Sigma) for 20 min followed by incubation with digoxigenin-labeled cRNA probes (DIG-11-UTP; Roche). Sense and anti-sense CXCL2 probes were transcribed from a 1.08-kb mouse CXCL2 cDNA, a gift of Dr. Luc Vallières, Laval University [16]. Hybridization was performed at 55 °C in 50% formamide with a final concentration of 100–200 ng of DIG probe/mL [17]. Following stringency washes, hybridization signal was identified with α-Digoxigenin-AP (Roche). Colorimetric images were converted to red as positive signal and black as negative, and overlaid onto DAPI stain of in situ sections. Images were taken with a Nikon Eclipse Ti-U with a Nikon D5-U2 camera using NIS Elements software. Appropriate processing including image overlays and black level and brightness adjustments were performed in Adobe Photoshop CC2014 and applied equally to all samples and controls.

### CNS mononuclear cell isolation

CNS tissue was harvested and dissected into the spinal cord and brainstem. Each specimen was homogenized in 1 mL PBS containing a protease inhibitor cocktail (Roche) and centrifuged at 800×*g* for 10 min. Supernatants were stored at − 80 °C for subsequent chemokine analysis. Infiltrating cells were isolated over a 27% Percoll gradient. Cells were quantified with a Cellometer AutoT4 automated cell counter, excluding dead or dying cells with trypan blue (Nexcelom).

### Flow cytometry

For surface staining, cells were resuspended in PBS + 2% fetal bovine serum (FBS) containing Fc Block (50 ng/mL) and Fixable Viability Dye efluor 506 (eBioscience) prior to incubation with fluorochrome-conjugated antibodies. For intracellular staining, cells were incubated with Brefeldin A (10 μg/mL) for 4 h in the presence or absence of stimulation conditions. Cells were labeled with fluorochrome-conjugated cell surface antibodies as described above, fixed in 4% PFA, permeabilized with 0.5% saponin, and incubated with fluorochrome-conjugated α-cytokine or chemokine antibodies. Stained cells were run on a FACS Canto II flow cytometer (v6.1.3, Becton Dickenson) or sorted on the FACS Aria II using FACS Diva software. Data was analyzed using FlowJo software (v10.0.7r2, Treestar).

### In vitro monocyte and neutrophil assays

Eight- to 14-week-old mice were euthanized; the femur and tibia were flushed and passed through a cell strainer (70 μm) with repeated washes. For monocyte stimulation, whole bone marrow was plated in complete media (RPMI with 10% FBS, L-Glutamine (2 mM, Gibco), Pen/Strep (1:100, Gibco), sodium pyruvate (12.5 μM, Gibco), and 2-mercaptoethanol (55 μm, Gibco)) in the presence of Brefeldin A (10 μg/mL) with lipopolysaccharide (LPS) (1 μg/mL), with or without IFNγ (2 ng/mL). Four hours later, the cells were stained for intracellular CXCL2 and subjected to flow cytometric analysis. Neutrophils were purified from bone marrow cell suspensions using an α-Ly6G microbead kit (Miltenyi). Purified neutrophils were plated in complete media in the presence or absence of CXCL2 (20 ng/mL), CXCL1 (20 ng/mL), granulocyte colony-stimulating factor (G-CSF) (25 ng/mL), IFNγ (2 ng/mL), or IL-1β (10 ng/mL) and isolated following 1 h in culture to examine CXCL2 mRNA expression.

### RNA isolation

Cells from FACS or in vitro cell culture were spun down and resuspended in 1 mL Trizol (Life Technologies). For RNA extraction, 200 μL of chloroform was added to samples and mixed prior to centrifugation at 18000×*g*. Chloroform layer was moved to a fresh tube with 500 μL cold isopropanol, mixed and incubated for 15 min prior to purifying the RNA out using the RNeasy MiniKit (Qiagen) with column DNase digestion per manufacturer’s instructions.

### RT- and q-PCR

RT-PCR was performed using the High Capacity cDNA Reverse Transcription Kit (Applied Biosystems) per manufacturer’s instructions. For q-PCR, TaqMan Universal Master Mix and primer/probe sets for CXCL2, CXCR2, and GAPDH were purchased from Applied Biosystems and run on a MyIQ system using iQ5 software (BioRad) as described in manufacturer’s instructions.

### Statistical analysis

Statistical analyses were performed using GraphPad Prism software. Unless otherwise stated, all graphs are expressed as means ± SEM. Comparisons of cell numbers, or of transcript levels in myeloid cells isolated from WT versus IFNγRKO hosts, were done using an unpaired Student’s *t* test. Comparisons of transcript levels in paired neutrophils or monocytes/macrophages isolated from mixed BM chimeras, or in myeloid cells cultured in vitro under different conditions, were done using a paired Student’s *t* test. A *p* value < 0.05 (*) was considered significant. *p* < 0.01 (**) and *p* < 0.001 (***).

## Results

### Inflammatory cells accumulate in the peri-pontine meninges during the early stages of both cEAE and aEAE, but only infiltrate the brainstem parenchyma of mice with aEAE

Our laboratory previously reported that neutrophils are more plentiful in the brainstems of IFNγRKO adoptive transfer recipients at the peak of aEAE compared with WT recipients at the peak of cEAE [5]. In contrast, at disease onset we found comparable numbers of brainstem-infiltrating CD11b^+^Ly6G^+^ neutrophils, as well as CD11b^+^Ly6G^−^ monocytes/macrophages, in IFNγRKO and WT recipients (Additional file 1: Figure S1). Immunohistochemical studies demonstrated that infiltrating inflammatory cells penetrate deep into the brainstem parenchyma at aEAE onset but are confined to the peri-pontine meninges at cEAE onset (Additional file 2: Figure S2). We detected T cells and neutrophils adjacent to the lateral recess of the fourth ventricle in every specimen examined, suggesting that leukocytes generally enter the brainstem via the choroid plexus, as previously reported in active immunization models [18]. We concluded that IFNγR signaling does not impede the early passage of inflammatory cells into the choroid plexus of adoptive transfer recipients, or their migration from the choroid plexus into the fourth ventricle and meninges, but suppresses their further extension into, and subsequent persistence in, the adjacent white matter parenchyma. As opposed to the anti-inflammatory role of IFNγ signaling in the brainstem, it is required for spinal cord infiltration since we find few inflammatory cells in the cords of IFNγRKO, as opposed to WT, adoptive transfer recipients at any time point following adoptive transfer [5].

### IFNγ suppresses CXCL2 expression by myeloid cells in the brainstem during cEAE

**Fig. 1:**
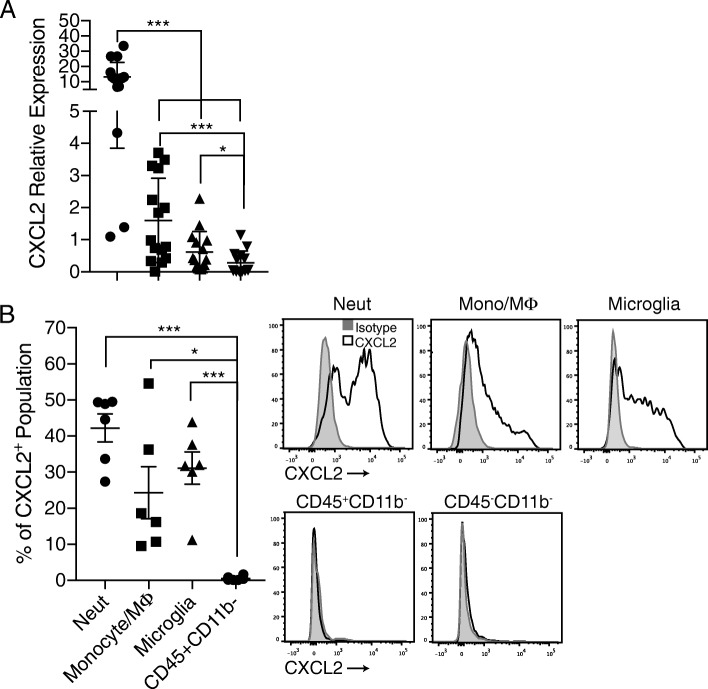
CD45^+^CD11b^+^ myeloid cells are a primary source of CXCL2 in the brainstem during aEAE. **a** Neutrophils (CD45^+^CD11b^+^Ly6G^+^), monocytes/macrophages (CD45^hi^CD11b^+^Ly6G^−^), microglia (CD45^lo^CD11b^+^Ly6G^−^), and non-myeloid immune cells (CD45^+^CD11b^−^) were FACS sorted from the brainstems of IFNγRKO mice at the onset of aEAE. CXCL2 mRNA was quantified by qPCR and normalized to GAPDH (*n* = 15 mice pooled from two experiments). **b** Brainstem-infiltrating cells isolated at the onset of aEAE were stained on the cell surface with antibodies specific for myeloid cell markers and intracellularly with αCXCL2 prior to flow cytometric analysis (*n* = 6 mice from two experiments)

The cellular subset that IFNγ modulates during cEAE to suppress parenchymal brainstem inflammation has not been definitively identified. MOG-specific Th1 cells induce clinical signs of aEAE at a twofold higher incidence in C57BL/6 conditional knockout mice that are IFNγR deficient in Lysozyme M (LysM)-expressing cells, when compared with their IFNγR-sufficient counterparts (Table 1). There was no difference between the groups in the incidence of clinical features characteristic of cEAE. This finding implicates myeloid cells as an important target of IFNγ in WT hosts. The ELR^+^ CXC chemokine, CXCL2, is elevated in the brainstem during aEAE and plays a critical role in the development of ataxia [5, 19]. We questioned whether CNS myeloid cells are the primary cellular source of CXCL2 in IFNγRKO transfer recipients. Indeed, CXCL2 mRNA was expressed at high levels in infiltrating monocytes/macrophages and neutrophils, as well as in microglia, FACS sorted from brainstems at the initiation of aEAE (Fig. 1a). CXCL2 mRNA was barely detectable above background levels in CNS-infiltrating CD45^+^CD11b^−^ cells, a cell surface phenotype consistent with lymphocytes. Intracellular staining and flow cytometric analysis demonstrated that neutrophils are the predominant producers of CXCL2 protein among CNS inflammatory cells in mice with aEAE (in terms of frequency of cells within the CXCL2^+^ population), followed by microglia and macrophages/monocytes (Fig. 1b). We did not detect CXCL2 protein in CNS CD45^+^CD11b^−^ lymphoid cells or in CD45^−^ non-hematopoietic cells. CXCL1, another member of the ELR+ CXC chemokine family, was not detectable in any of the inflammatory cell subsets (data not shown).

**Table 1:** IFNgR deficiency in Lysozyme M (LysM)-expressing myeloid cells increases susceptibility to EAE

Genotype	Incidence of aEAE	Average cumulative clinical score aEAE	Incidence of cEAE	Average cumulative clinical score cEAE
Cre−	4/13 (30.8%)	10.5 ± 1.11	9/13 (69.2%)	26.7 ± 1.93
Cre+	16/26 (61.5%)	6.44 ± 0.226	18/26 (69.2%)	32.4 ± 1.04

LysM-Cre:IFNgR^flox/flox^ mice and their non-Cre expressing littermates were injected with MOG_35–55_-reactive encephalitogenic Th1 cells. Mice were evaluated for signs of aEAE or cEAE on a daily basis by examiners blinded to group identity

**Fig. 2:**
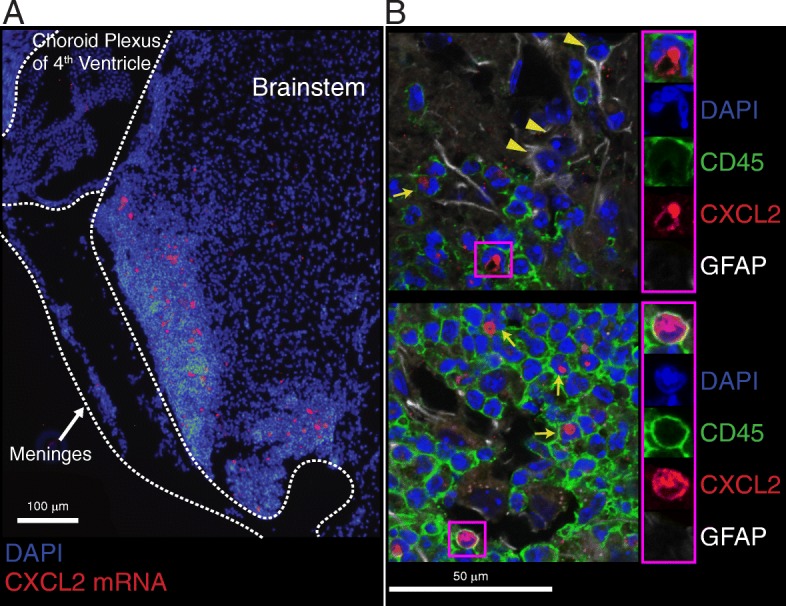
CXCL2 is produced by CD45^+^ cells in the brainstem infiltrates during aEAE. **a** Brainstem tissue was harvested from an IFNγRKO host at aEAE onset (clinical score 1). Sections were subjected to in situ hybridization to detect CXCL2 mRNA (red) combined with a DAPI (blue) counter stain. **b** Brainstem sections from a mouse with aEAE (clinical score 3) were stained with monoclonal antibodies specific for CD45 (green), GFAP (white), and CXCL2 (red) and counterstained with DAPI (blue). Arrows indicate CXCL2^+^ CD45^+^ cells. Arrow heads indicate GFAP^+^ astrocytes

Consistent with the above findings, CXCL2 was expressed within intraparenchymal inflammatory infiltrates in brainstem sections obtained at peak aEAE, but not in adjacent uninflamed white matter or in the meninges (Fig. 2a). In immunohistochemical studies, the CXCL2 producers were exclusively CD45^+^ hematopoietic cells (Fig. 2b; Additional file 3: Figure S3). Many CD45^+^ cells that stained positively for CXCL2 had multilobulated nuclei, indicative of neutrophils. GFAP-positive astrocytes, present at the border of the inflammatory infiltrates, were CXCL2 negative. We did not detect CXCL2 expression in the brainstem of WT hosts with cEAE, either by in situ hybridization or immunohistochemistry (unpublished data).

### IFNγ suppresses CXCL2 in brainstem myeloid cells via a cell intrinsic pathway

**Fig. 3:**
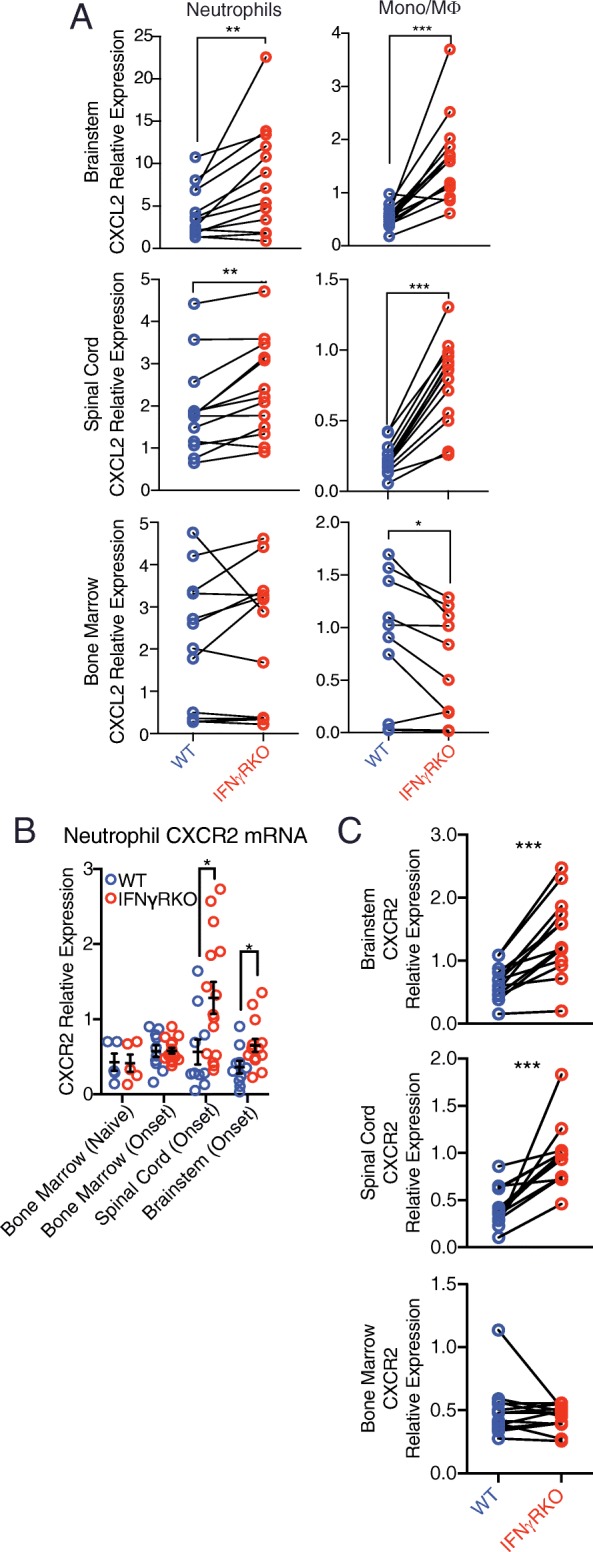
IFNγ signaling directly regulates CXCR2 expression by neutrophils. **a** IFNγR-sufficient and IFNγRKO neutrophils (left panels) and monocytes/macrophages (right panels) were FACS sorted from the brainstems (upper panels), spinal cords (middle panels), and bone marrow (lower panels) of mixed bone marrow chimeric mice. CXCL2 transcript levels were measured by qPCR and normalized to GAPDH (*n* = 13 chimeric mice from two experiments). **b** CD45^+^CD11b^+^Ly6G^+^ neutrophils were FACS sorted from the spinal cord, brainstem, and bone marrow of WT mice at the onset of cEAE, and IFNγRKO mice at the onset of aEAE. Bone marrow cells from naïve mice were used as controls. CXCR2 mRNA levels were quantified by qPCR and normalized to GAPDH (*n* = 10 WT mice and 15 IFNγRKO mice with EAE, pooled from two experiments; *n* = 4 WT naive mice and 5 IFNγRKO naïve mice). **c** IFNγR-sufficient and IFNγRKO neutrophils (defined as CD45^+^CD11b^+^Ly6G^+^) were FACS sorted from the bone marrow, spinal cord, and brainstem of mixed bone marrow chimeric mice at the onset of EAE. CXCR2 transcript levels were normalized to GAPDH (*n* = 13 mice from two experiments)

To investigate whether IFNγ directly suppresses CXCL2 production in CNS myeloid cells during EAE, we constructed mixed bone marrow chimeric mice by reconstituting lethally irradiated CD45.1 congenic C57BL/6 hosts with a mixture of bone marrow cells from IFNγR-sufficient and IFNγRKO donors. IFNγR-sufficient donor cells were distinguished by expression of a tdTomato reporter gene. Eight weeks later, the reconstituted chimeras were injected with MOG-reactive Th1 cells in order to induce EAE. The brainstem, spinal cord, and bone marrow cells were harvested at EAE onset, and myeloid cell subsets from each donor pool were subjected to qPCR. CXCL2 transcript levels were consistently reduced in IFNγR-sufficient myeloid cells compared with paired IFNγRKO myeloid cells isolated from the CNS of individual chimeric mice (Fig. 3a). In contrast, CXCL2 mRNA expression was comparable in IFNγR-sufficient versus IFNγRKO bone marrow neutrophils, and slightly lower in IFNγRKO bone marrow monocytes.

### IFNγ suppresses expression of CXCR2 by brainstem-infiltrating neutrophils

In a model of *Listeria monocytogenes* infection, IFNγ downregulates expression of CXCR2 (the only receptor for CXCL2 in mice) on neutrophils, thereby curtailing their recruitment to the site of inflammation [20]. We questioned whether IFNγ similarly suppresses CXCR2 expression on neutrophils during early stages of cEAE. CXCR2 is rapidly internalized following ligation by its cognate chemokine, such that cell surface CXCR2 levels are not always a reliable indicator of CXCR2 expression. Therefore, we chose to measure CXCR2 transcripts in neutrophils harvested from mice with aEAE or cEAE by qPCR. CXCR2 transcript levels were significantly higher in neutrophils isolated from the brainstems and spinal cords of IFNγRKO hosts at aEAE onset compared with analogous neutrophils isolated from WT hosts at cEAE onset (Fig. 3b). There was no difference in CXCR2 expression in neutrophils isolated from the bone marrow of IFNγRKO versus WT hosts at the same time point, or from naïve IFNγRKO versus WT mice. CXCR2 mRNA was not expressed at detectable levels by microglia, or CNS-infiltrating monocytes or lymphocytes, that were FACS sorted from mice during either aEAE or cEAE (data not shown).

We next compared CXCR2 mRNA expression in IFNγ-responsive versus unresponsive neutrophils isolated from the brainstems or spinal cords of mixed bone marrow chimeric mice with EAE, using the approach described in Fig. 3a. CXCR2 mRNA levels were reproducibly higher in IFNγRKO CNS-infiltrating neutrophils compared to paired IFNγR-sufficient CNS-infiltrating neutrophils isolated from individual mice (Fig. 3c). In contrast, there was no difference in CXCR2 mRNA expression when comparing IFNγR-sufficient versus IFNγRKO bone marrow neutrophils from the same mice. This indicates that during EAE, IFNγ directly inhibits CXCR2, as well as CXCL2, production by IFNγ-responsive neutrophils, specifically within the CNS microenvironment.

### IFNγ regulates a CXCL2 autocrine feedback loop in neutrophils

**Fig. 4:**
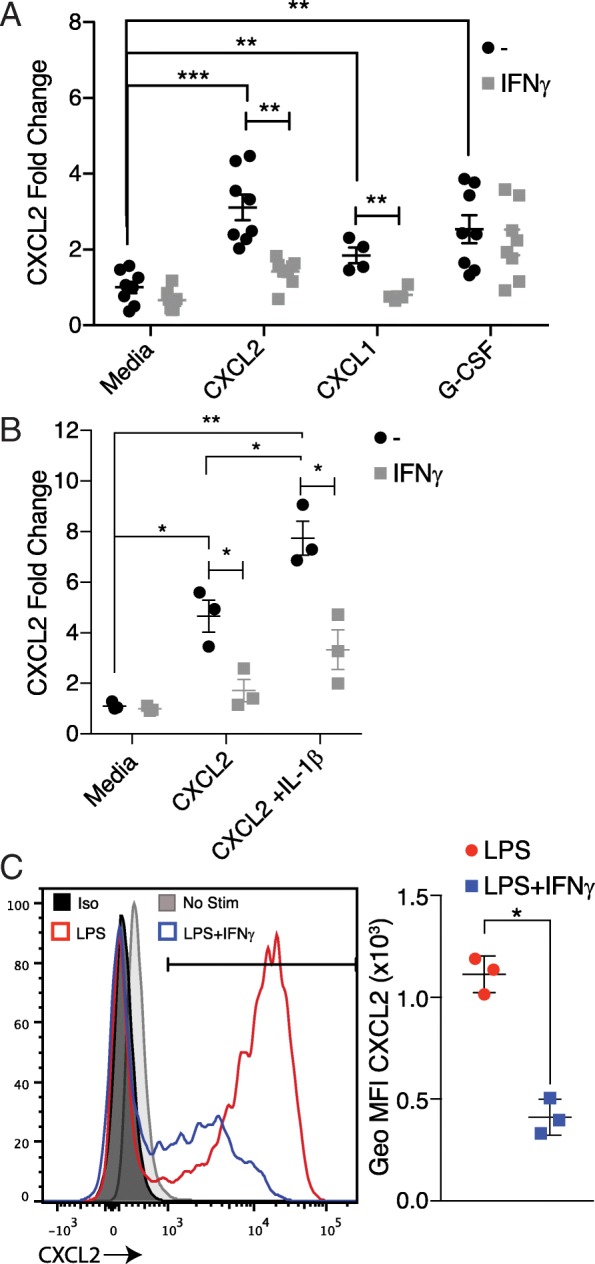
IFNγ suppresses CXCL2 expression in neutrophils and monocytes stimulated with CXCR2 or TLR ligands, respectively. **a**, **b** Bone marrow neutrophils were cultured in media alone, or with recombinant CXCL2, CXCL2 plus IL-1β, CXCL1, or G-CSF, in the presence or absence of IFNγ. RNA was extracted 1 h later to measure CXCL2 transcript levels via qPCR. Fold induction of CXCL2 in the stimulated neutrophils over unstimulated controls was calculated as 2^−ΔΔCt^, using GAPDH as an internal control for normalization. Each symbol represents a neutrophil culture derived from an individual mouse. (**a** *n* = 8 mice, with data pooled from three experiments. **b** Representative of two experiments with *n* = 3 mice each). **c** Bone marrow monocytes were stimulated with LPS in the presence or absence of IFNγ for 4 h. Intracellular CXCL2 protein expression was assessed by flow cytometry (Representative of three experiments with *n* = 3 mice each)

IL-17 is a potent inducer of ELR^+^ CXC chemokines and plays a critical role in initiating neutrophil migration to the brainstem white matter in some models of aEAE [13, 19]. However, the model of aEAE employed in the present study is IL-17 independent [5]. This raises the question of which factors actively drive CXCL2 production in the brainstem of C57BL/6 IFNγRKO mice following the adoptive transfer of MOG_35–55_-reactive CD4+ Th1 cells. To address that issue, we screened soluble factors, known to be upregulated in the brainstems over the spinal cords of mice with aEAE [5], for their ability to stimulate CXCL2 production in naïve C57BL/6 bone marrow neutrophils in vitro. CXCL2 mRNA was upregulated in purified neutrophils following culture either with recombinant G-CSF, CXCL1, or CXCL2 itself (Fig. 4a). In contrast, neither recombinant CCL3 nor CCL4 altered the expression of CXCL2 transcripts in cultured neutrophils (data not shown). Furthermore, neutrophil CXCR2 expression was not affected by culture with any of the recombinant chemokines. Induction of CXCL2 mRNA in neutrophils cultured with recombinant CXCL2 was enhanced by IL-1β (Fig. 4b). IFNγ suppressed CXCL2 mRNA upregulation in neutrophils cultured with CXCL2, either alone or combined with IL-1β, but had no effect on CXCL2 mRNA levels in G-CSF-stimulated neutrophils (Fig. 4a, b). Ligation of toll-like receptors (TLRs) on monocytes, macrophages, and microglia is known to upregulate CXCL2 expression [21, 22]. To examine whether CXCL2 expression in non-granulocytic myeloid cells could be regulated by IFNγ, we cultured bone marrow-derived monocytes with LPS in the presence or absence of IFNγ. LPS triggered robust CXCL2 expression in bone marrow monocytes, which was inhibited by IFNγ (Fig. 4c).

### CXCL1 is expressed by non-hematopoietic CNS resident cells during EAE

**Fig. 5:**
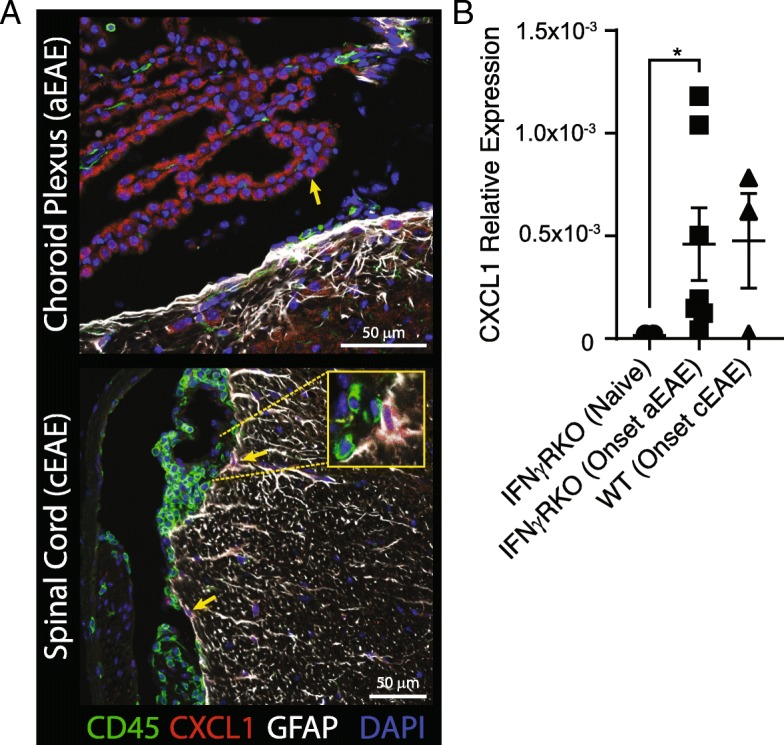
CXCL1 is expressed by CNS resident cells during EAE. **a** CXCL1 (red) was detected in the choroid plexus epithelium in brainstem sections of IFNγRKO mice at the onset of aEAE (clinical score 1), and in astrocytes in spinal cord sections of WT mice at the onset of cEAE (clinical score 2), via immunofluorescent histology. Sections were co-stained with antibodies specific for CD45 (green) and GFAP (white), and counterstained with DAPI (blue). **b** Choroid plexus samples were obtained at the onset of cEAE or aEAE, or from naïve IFNγRKO mice. CXCL1 transcript was quantified by qPCR and normalized to GAPDH (*n* = 4–6/group from two experiments)

CXCL1 and CXCL2 are both ligands for CXCR2. In a previous study, we made the paradoxical observation that although CXCL2 is disproportionately upregulated in the brainstem of IFNγRKO mice with aEAE in comparison to WT mice with cEAE, CXCL1 expression is actually expressed at elevated levels in the brainstem and spinal cord of the WT adoptive transfer recipients [5]. Our discovery that IFNγ downregulates CXCR2 on neutrophils (Figs. 3 and 4) provides one explanation for why CXCL1 is unable to compensate for low CNS CXCL2 levels in WT adoptive transfer recipients and promote parenchymal brainstem infiltration. A complementary hypothetical explanation is that CXCL1 is produced by a different cell type in the brainstem than CXCL2, at a location that does not facilitate migration into the white matter. Indeed, immunohistochemical analyses showed that the choroid plexus is a major source of CXCL1 during EAE in WT, as well as IFNγRKO, mice (Fig. 5a and data not shown). This observation was corroborated by qPCR, which revealed an upregulation of CXCL1 transcripts in choroid plexus epithelial cells isolated from mice with either cEAE or aEAE (Fig. 5b and data not shown). We also detected CXCL1 in astrocytes in the spinal cords of WT hosts with cEAE (Fig. 5a). We did not detect CXCL1 in infiltrating inflammatory cells, irrespective of CNS compartment or host genotype. These results corroborate published data that demonstrates CXCL1 upregulation in the choroid plexus of C57BL/6 mice with EAE induced by active immunization [16].

## Discussion

Based on the results of this study, we propose the following hypothetical model by which an IFNγ/CXCL2 axis regulates the regional localization of inflammatory infiltrates during EAE. CXCL1 is released by choroid plexus epithelium in the early stages of both cEAE and aEAE, and promotes neutrophil migration into the fourth ventricle and meningeal space. In IFNγRKO hosts, the neutrophils are then stimulated to migrate deeper into the brainstem parenchyma by a CXCL2 concentration gradient, initially generated by activated microglia, and subsequently amplified by neutrophils and monocytes as they accumulate in the brainstem white matter. Astrocyte-derived CXCL1 might also contribute to the early infiltration of the brainstem parenchyma by neutrophils. CXCL2 production by CNS myeloid cells escalates during the progression of aEAE, driven by an autocrine and/or paracrine positive feedback loop (as suggested by the data in Fig. 4). Conversely, in WT hosts, CXCR2 and CXCL2 expression in CNS myeloid cell populations is suppressed by IFNγ that is released by reactivated myelin-reactive T cells. Consequently, neutrophils do not penetrate deep into the brainstem white matter. The inhibition of CXCR2 and CXCL2 expression by CNS-infiltrating neutrophils in response to IFNγ signaling appears to be a cell- and microenvironment-specific phenomenon, since IFNγ has been reported to actually promote CXCR2 and CXCL2 production by non-hematopoietic cells in non-CNS tissues [23–25]. Reminiscent of the results of the current study, infiltrating neutrophils were recently found to be the major source of CXCL2 in a mouse model of immune complex-mediated cutaneous inflammation (ICMCI) [26]. Expression of CXCL2 by immune complex-activated neutrophils amplified their recruitment to the skin in an autocrine/paracrine manner. The ICMCI model also resembles our Th1-mediated EAE model in that tissue resident cells were the major source of CXCL1. In both animal models, differences in the cellular source and spatial distribution of CXCL1 and CXCL2 translate into distinct roles of those chemokines in the pathogenic process.

Although the model of Th1-mediated aEAE described here is IL-17 independent, that is not the case for an alternative aEAE model induced in C3Heb/Fej mice by the adoptive transfer of IL-23 polarized, MOG_97–114_-reactive CD4^+^ Th17 cells [13, 19]. The latter model of aEAE, like ours, is neutrophil dependent and associated with CXCL2 induction in the brain [19]. Astrocytes isolated from the brain and spinal cord of C3Heb/Fej mice at peak aEAE express higher levels of CXCL2 mRNA than endothelial cells, microglia, or CNS-infiltrating myeloid cells [19]. In contrast, infiltrating myeloid cells and microglia are a major source of CXCL2 in the brainstem of C57BL/6 IFNγRKO recipients following the transfer of encephalitogenic Th1 cells (Figs. 1 and 2). CXCL2 itself, in combination with IL-1β, is a likely candidate for the inducer CXCL2 in our model (Fig. 4). We did not detect CXCL2 in GFAP^+^ astrocytes in brainstem sections of symptomatic C57BL/6 IFNγRKO hosts. However, GFAP staining was relatively sparse in our brainstem sections. In addition, astrocytes were rare among the CD45^−^ cells that we isolated from brainstem preparations for flow cytometric analysis (data not shown). Therefore, we cannot rule out the possibility that, in our aEAE model, astrocytes and/or other non-hematopoeitic CNS resident cells contribute to brainstem CXCL2 expression in conjunction with myeloid cells. The contention that IFNγ suppresses aEAE via effects on multiple cellular targets in parallel is supported by the increased susceptibility of both IFNγRKO ➔WT and WT➔IFNγRKO bone marrow chimeric mice to aEAE [15].

The intracellular pathways that regulate neutrophil CXCL2 expression during aEAE remain to be elucidated. The fact that IFNγ inhibits CXCL2-induced, but not G-CSF-induced, upregulation of CXCL2 in cultured neutrophils (Fig. 4) suggests the co-existence of multiple, parallel pathways. LysM-Cre:SOCS3^fl/fl^ conditional knockout mice develop an atypical form of EAE that is ameliorated by neutrophil depletion or CXCR2 blockade [27]. Neutrophils from LysM-Cre:SOCS3^fl/fl^ mice exhibit enhanced and prolonged activation of STAT3 in response to stimuli such as G-CSF. We are currently investigating the role of a SOCS3/STAT3 immunoregulatory circuit in our model.

## Conclusions

The current study adds to a growing body of literature demonstrating diversity in the dysregulated immune pathways that drive, and modulate, CNS autoimmune disease. Clinical variants of human autoimmune demyelinating disease display different patterns of lesion distribution. For example, the spinal cord is disproportionately targeted in an opticospinal form of MS that is prevalent in Asia [28, 29]. In contrast, lesion burden is skewed towards the cerebrum in most individuals in the Western hemisphere with relapsing-remitting MS [30]. Differences in underlying immunopathological mechanisms and lesion distribution between MS patients could correlate with differences in environmental triggers, and ultimately translate into differences in therapeutic responsiveness to individual disease-modifying drugs [31–33]. Collectively, the current study, together with previously published studies in aEAE and cEAE, suggests that actionable therapeutic targets may differ among MS patients with distinct types of immune dysregulation and clinical phenotypes. Conversely, in some cases the role of a particular mediator and/or leukocyte might be universal across individuals within the same clinical subset (as with the regulatory role of IFNγ in brainstem inflammation). These observations illustrate the complexity of the relationship between immunopathological pathways and their clinical manifestations. They underscore the importance of discovering biomarkers that correlate with underlying immune mechanisms, in addition to clinical phenomenology, to help guide the management of inflammatory demyelinating disease in a customized manner.

## Additional files

Additional file 1: **Figure S1.** The numbers of CNS-infiltrating myeloid cells are comparable at the onset of cEAE and aEAE. Spinal cords (left panels) and brainstems (right panels) were harvested at the onset of cEAE in WT adoptive transfer recipients (blue) or at the onset of aEAE in IFNγRKO adoptive transfer recipients (red). Inflammatory cells were isolated and analyzed by flow cytometry. The numbers of CD45^hi^CD11b^+^Ly6G^−^ monocytes/macrophages (upper panels) and CD45^hi^CD11b^+^Ly6G^+^ neutrophils (lower panels) were counted per specimen. Each symbol reflects the results obtained from an individual mouse. (PDF 694 kb)

Additional file 2: **Figure S2.** Neutrophils only infiltrate the brainstem white matter parenchyma during aEAE. Immunofluorescent histology was performed on brainstem sections obtained at the onset of cEAE (left panels) or aEAE (right panels) to detect cells expressing CD3ε (green), Ly6G (red), and DAPI (blue). (PNG 1607 kb)

Additional file 3: **Figure S3.** Expression of CXCR2 is not altered produced by CD45^+^ cells in the brainstem infiltrates during aEAE. Images of the brainstem section, shown in Fig. 2b, using individual fluorescent channels. (PDF 3879 kb)

## Abbreviations

aEAE: Atypical EAE
cEAE: Conventional EAE
CNS: Central nervous system
EAE: Experimental autoimmune encephalomyelitis
FACS: Fluorescence-activated cell sorting
FBS: Fetal bovine serum
G-CSF: Granulocyte colony-stimulating factor
GFAP: Glial fibrillary acidic protein
IFNγR: IFNγ receptor
LPS: Lipopolysaccharide
qPCR: Quantitative polymerase chain reaction
WT: Wildtype
