# Supplementary figures and images for: An IFNγ/CXCL2 regulatory pathway determines lesion localization during EAE
Source: J Neuroinflammation. 2018 Jul 16;15:208. doi: 10.1186/s12974-018-1237-y (PMC6048869; doi:10.1186/s12974-018-1237-y)

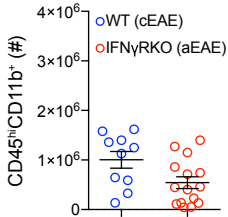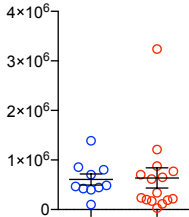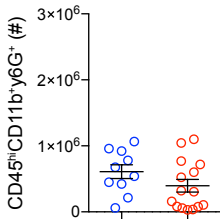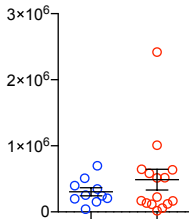

Supplement: Supplementary file 1 — Figure S1. The numbers of CNS-infiltrating myeloid cells are comparable at the onset of cEAE and aEAE. Spinal cords (left panels) and brainstems (right panels) were harvested at the onset of cEAE in WT adoptive transfer recipients (blue) or at the onset of aEAE in IFNγRKO adoptive transfer recipients (red). Inflammatory cells were isolated and analyzed by flow cytometry. The numbers of CD45hiCD11b+Ly6G− monocytes/macrophages (upper panels) and CD45hiCD11b+Ly6G+ neutrophils (lower panels) were counted per specimen. Each symbol reflects the results obtained from an individual mouse. (PDF 694 kb) [file 12974_2018_1237_MOESM1_ESM.pdf]

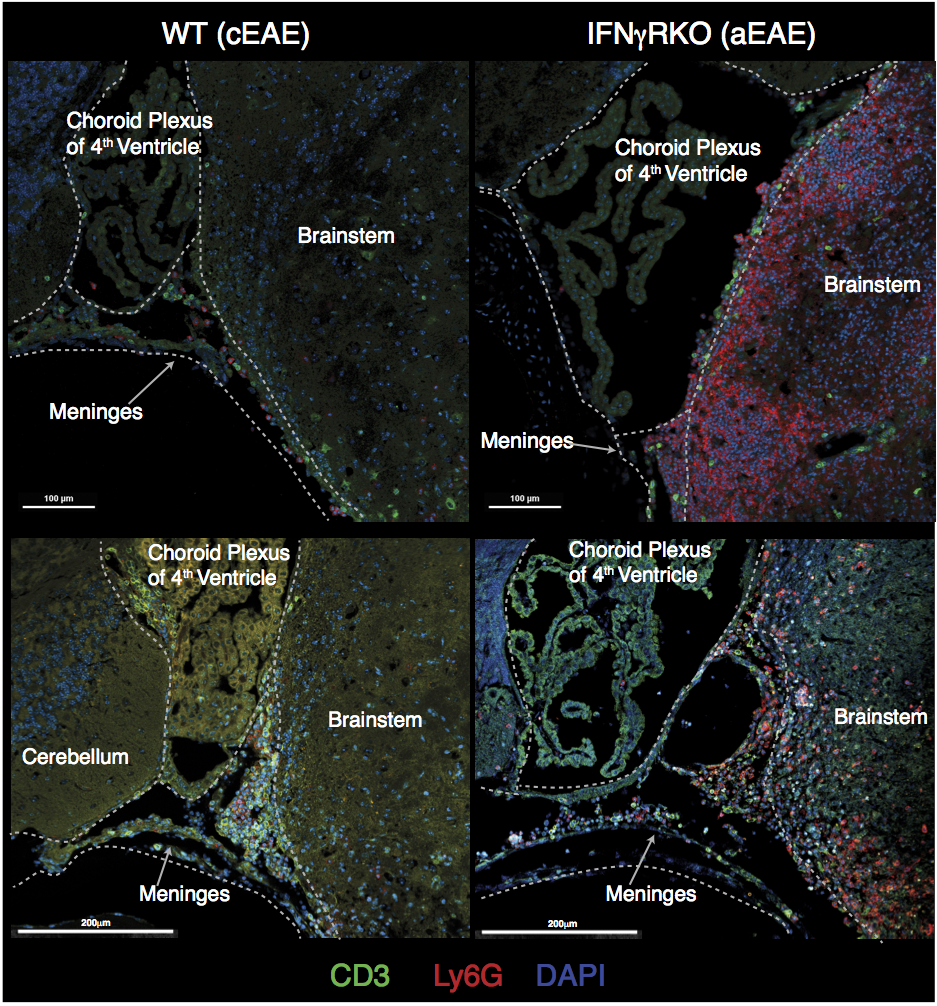

Supplement: Supplementary file 2 — Figure S2. Neutrophils only infiltrate the brainstem white matter parenchyma during aEAE. Immunofluorescent histology was performed on brainstem sections obtained at the onset of cEAE (left panels) or aEAE (right panels) to detect cells expressing CD3ε (green), Ly6G (red), and DAPI (blue). (PNG 1607 kb) [file 12974_2018_1237_MOESM2_ESM.png]

A

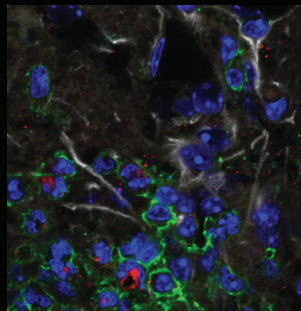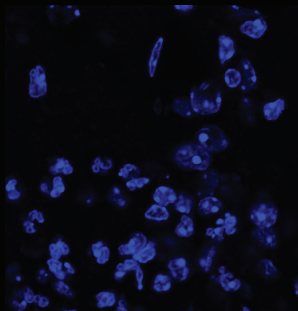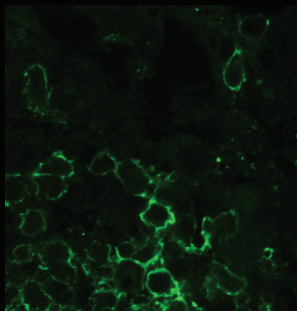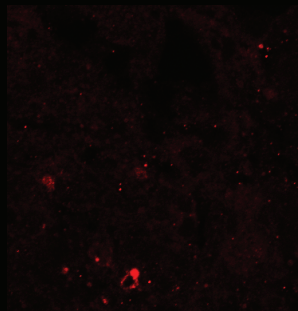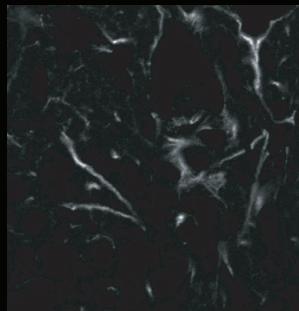

B

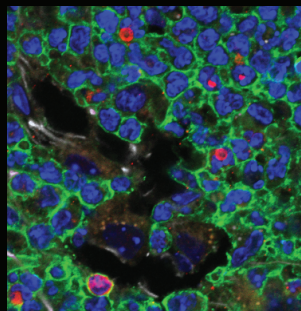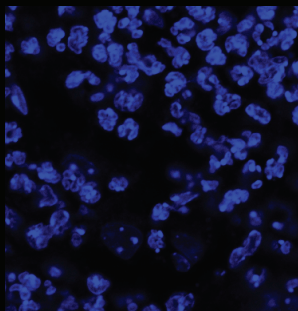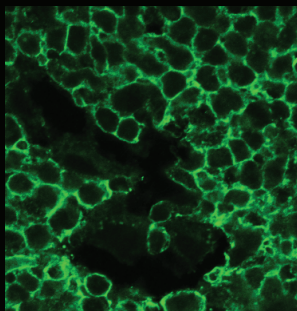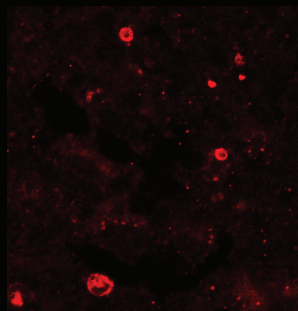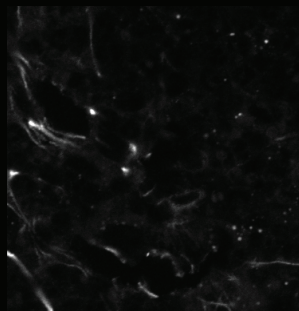

DAPI

CD45

CXCL2

GFAP

Supplement: Supplementary file 3 — Figure S3. Expression of CXCR2 is not altered produced by CD45+ cells in the brainstem infiltrates during aEAE. Images of the brainstem section, shown in Fig. 2b, using individual fluorescent channels. (PDF 3879 kb) [file 12974_2018_1237_MOESM3_ESM.pdf]
